# The food of life: an evaluation of the impact of cash grant receipt and good parenting on child nutrition outcomes in South Africa and Malawi

**DOI:** 10.1177/1757975920957598

**Published:** 2020-09-30

**Authors:** Lorraine Sherr, Kathryn J. Roberts, Helen Mebrahtu, Mark Tomlinson, Sarah Skeen, Lucie D. Cluver

**Affiliations:** 1 Institute for Global Health, University College London, London, UK; 2 Institute for Life Course Health Research, Department of Global Health, Stellenbosch University, Stellenbosch, South Africa; 3 School of Nursing and Midwifery, Queens University, Belfast, UK; 4 Centre for Evidence-Based Intervention, Department of Social Policy & Intervention, University of Oxford, Oxford, UK; 5 Department of Psychiatry and Mental Health, University of Cape Town, Cape Town, South Africa

**Keywords:** Cash transfer, care, good parenting, social protections, nutrition, South Africa, Malawi

## Abstract

Social protection interventions (inclusive of cash grant receipt and care provision) have been found to be effective in response to some of the negative implications of the HIV epidemic on children and families. This study explores the impact of cash grant receipt and care provision (operationalised as good parenting) on child nutritional outcomes. In this cross-sectional study, 854 children and younger adolescents (5–15 years) and caregivers affected by HIV, attending community-based organisations in South Africa and Malawi, were interviewed. Interviews comprised inventories on socio-demographic information, family data, cash grant receipt and child nutrition. Parenting was measured using a composite scale. Logistic regression and marginal effects analyses were used to explore the associations between differing levels of social protection (none; either cash or good parenting; cash and good parenting) and child nutritional outcomes. One hundred and sixty children (20.3%) received neither cash nor good parenting; 501 (63.5%) received either cash or good parenting and 128 (16.2%) received both cash and good parenting. In comparison to no intervention, receipt of either cash or good parenting was significantly associated with child non-stunting, the child having sufficient food, and the child not looking thin. Three (3/7) nutritional outcomes showed increased improvement amongst children receiving both cash and good parenting care including child-reported non-hunger, child non-stunting and parental report of sufficient food. Marginal effects analyses further identified an additive effect of cash and good parenting on child nutritional outcomes. This study indicates that receipt of combined cash and good parenting, when compared to cash grant receipt alone, has positive effects on nutrition-related child outcomes.

## Introduction

Food and nutrition play a key role in the complex impact of HIV infection at the household level (1). Patenaude *et al.* (1) provided evidence that antiretroviral treatment (ART) commencement had a distinct impact on household food security, noting that ART commencement was associated with both adult and child missed meals. Although this study could not clearly identify the mechanism for food insecurity, the authors call for policy consideration to redress this issue (1). Such policy could potentially include cash grants to the household, or the availability of quality care provision, both of which have been shown to improve child outcomes generally, and nutrition as well as child development specifically. The recent sustainable development goals (SDGs) have indicated that ending hunger (SDG2) is of fundamental importance to long-term human development.

There is solid evidence that child development can be negatively affected by stunting and malnutrition (2,3). The effects of malnutrition can be long-lasting (4). When food insecurity and HIV co-exist, the effects on both child development and child behaviour can be dramatic (5). HIV has implications for child and adolescent development. This is true for children and adolescents living with HIV, HIV-exposed uninfected children and adolescents, and for children and adolescents living in households where at least one adult is infected with HIV – such associations are often exacerbated by poverty, unemployment, parenting challenges and food insecurity (6). However, there is an absence of studies investigating the developmental and behavioural outcomes of insufficient nutrition in sub-Saharan Africa where there is often high rates of poverty, nutritional challenge and co-occurring HIV infection amongst children and families. Likewise, there remains a paucity of literature addressing food insecurity of children and adolescents infected with or affected by HIV; a systematic review of interventions for severe acute malnutrition in young children identified 68 studies on the topic (7) – none of which covered children infected or affected by HIV.

Cash transfers as a form of social protection have been proposed as a highly effective intervention to alleviate poverty and to reduce child and adolescent exposure to risk. Amongst adolescents, where there is considerable HIV risk behaviour associated with poverty (such as transactional sex), analyses have shown that cash transfers can break the cycle of such risk behaviours. Furthermore, when care is provided in addition to cash transfers, this more robust combination package has been found to have increased effectiveness in relation to adolescent risk behaviour (8). Within this study by Cluver *et al.* (8), the outcome measures of interest were adolescent sexual risk behaviours (i.e. transactional sex, age-disparate sex, early sexual debut, and condomless sex). When it comes to younger children and young adolescents, such outcomes are not yet an issue. Predictors of adolescent risk-taking have been shown to be linked to a variety of factors including cognitive development, educational risk and poverty (and thus by proxy poor nutrition) (9). Such exposures may be on the pathway to risk, and early intervention for children and younger adolescents may avoid such risk pathways.

Cash transfers have been specifically evaluated as an intervention to improve nutrition outcomes, with mixed results (10,11). A trial conducted in Burkina Faso, found that seasonal transfers did not result in a significant decrease in malnutrition as such; the authors suggested the need to examine complementary interventions in the pursuit of improved nutritional outcomes (10,12). However, a similar intervention in Niger was found to be effective (13). Various forms of cash transfer provision have been studied in terms of impact on different nutrition outcomes (including wasting, stunting, height-based growth, food security, hunger) and at different stages (14). Timing and amount of cash transfers have been shown to be important variables. The evidence regarding the impact of conditional cash transfers on child nutritional outcomes within sub-Saharan Africa is beginning to be summarised (15). However, there is now a need to examine combination interventions within a broader social protection paradigm, to identify specific combinations of social protection that provide maximum traction for improved child nutritional outcomes. Cash transfers, and cash plus good-quality care have been shown to be related to educational risk reduction and positive cognitive development in childhood in studies of HIV-infected and -affected groups (16,17). Yet to date, there is no single study that has explored the impact of cash and cash plus care on nutrition outcomes for children and younger adolescents in sub-Saharan Africa. Cash transfers are seemingly most effective when they form part of a complex basket of provision for individuals. Supplements to cash have been studied to include good parenting (16,17), good clinic care, and support (18,19). This study explores the effect of cash transfers and combined cash receipt and care provision (operationalised as good parenting) on child and young adolescent (5–15 years) nutritional outcomes.

## Methods

### Procedure

In this cross-sectional study, consecutive child and young adolescent attenders (aged 5–15 years) and their primary caregivers were interviewed independently by trained data collectors using questionnaires administered using mobile phone technology (20). Full study information was provided. Informed written consent was obtained from all primary caregivers, and assent from all children within the study. Questionnaires, for both children and caregivers, included a range of study-specific questionnaires and standardised measures relating to health, wellbeing, cognition, nutrition and socio-demographic information. All study information, consent forms and questionnaires were translated into Zulu Xhosa and Chewe as appropriate and back translated for administration.

### Measures

#### Socio-demographic characteristics

Demographic characteristics (child/adolescent age, gender, HIV status) were obtained using caregiver reports. The type of household that the child/adolescent lived in was also obtained using caregiver reports (i.e. house/flat, a shack, on the street), and responses were dichotomised into formal (house/flat) versus informal (shack/street) housing. Household wealth was assessed using an item from the Demographic and Health Survey (DHS) focusing on the number of household assets (21). Caregivers were asked to identify how many household items they owned: refrigerator, stove, television, radio, telephone, mobile phone, computer, internet, car, and bicycle. Number of assets were scored on a scale between 0 and 10 (scoring 1 point for each asset owned), with higher scores representing a greater number of assets.

#### Cash grant receipt

Grant receipt was determined by caregiver reports. Caregivers were asked whether they received any of the following grants into the household: state pension, retirement pension, disability grant, child support grant, foster care grant, care dependency grant or any other cash transfer support. Grant receipt was dichotomised with regard to whether any grant was received versus no grants received.

#### Care receipt: good parenting

Care within the context of this study was defined as ‘good parenting’. A measure of good parenting has been used within pre-existing studies associated with this data (12). The good parenting measure was operationalised based on a composite index of 10 items made up of both child/adolescent and caregiver responses. Children/adolescents within the study reported on four items drawn from the Child Status Index tool (22) including whether they received praise, whether they felt that they belonged in their home, whether they received treats and whether adults hugged children as well as praised them. Caregivers reported on six items drawn from the Parent–Child Conflict Tactics Scale (23) including provision regarding consistent care, the use of positive discipline (i.e. taking away privileges, explaining what children did wrong) and the absence of emotional or physical violence towards the child. All items were given a binary score (yes/no). The index was scored from zero to 10, with higher scores reflecting more positive parenting practices (12). The index was dichotomised within the study with a score of eight or above being identified as ‘good-enough parenting’ (*n* = 101) and those scoring below eight (*n* = 732). No participants scored 10 and only one participant scored nine, therefore eight was chosen as a cut-off to reflect a high enough standard of parenting.

#### Nutrition outcomes

Seven measures of nutrition were used as outcome measures and included both child/adolescent and caregiver reports. Children and young adolescents within the study reported whether they had gone to bed hungry the previous night, an item drawn from the Child Status Index tool (22). This item was given a binary score of yes/no. Measures of age, height and weight, were used to develop standardised World Health Organization measures of height-for-age, weight-for-age and weight-for-height. These items were used to assess malnutrition: whether children were stunted, wasted or underweight (<−2 *z*-score). These measures were given a binary score (i.e. yes – stunted, no – normal growth). Caregiver report was also used to establish child food status (whether the child has sufficient food all of the time, regularly, less food than needed, or regularly no food to eat) based on an item drawn from the Child Status Index (22). This item was dichotomised to distinguish sufficient food all of the time (*n* = 515) versus not (*n* = 339). Caregivers also reported on whether the child was small for their size and whether the child looked thin – items drawn from the nutrition and growth domain of the Information and Action Tool (24). These items were given a binary score (i.e. yes – child is thin, no – normal growth). All items were recoded to focus on positive nutritional outcomes, that is, Did you go to bed hungry last night? (1 = no, 0 = yes).

### Participants

The study sample included children and young adolescents (5–15 years; *n* = 854) and their primary caregivers (*n* = 854). Data collection was undertaken between 2013 and 2014 as part of the Child Community Care Study, which aims to track children and families affected by HIV who attend community-based organisations within South Africa and Malawi. Five hundred and eighty-eight community-based organisations (524 in South Africa and 64 Malawi) were identified as all funded child providers by 11 partner organisations (AIDS Alliance, Stop AIDS Now, Diana Memorial Fund, Firelight Foundation, Bernard van Leer foundation, REPSSI, World Vision, Comic Relief, Help Age, Save the Children and UNICEF). All 588 identified community-based organisations were stratified by geographical location and funding partners and 28 (24 in South Africa and 4 in Malawi) were randomly selected. All 28 community-based organisations agreed to participate. Ethical approval was obtained from University College London Research Ethics Committee (reference number 1478/002) and Stellenbosch University Health Research Ethics Committee (reference number N10/04/112) and authorised by each of the funding partners of the various community-based programmes in each respective country.

### Statistical analyses

All analyses were carried out using Stata v.13 (25). Differences between those who received a household cash grant and those who did not receive any grant were explored with regard to socio-demographic characteristics inclusive of seven measures of child nutritional status using chi-square and *t*-tests. Results are reported using mean and standard deviations (SD) for continuous variables, and frequency percentages for categorical variables. A series of logistic regression models were used to examine the associations of cash or parenting support and combined support (represented by indicator variables using no support as the reference category) with nutritional outcomes. Model 1 shows the unadjusted univariate associations between cash and parenting support and nutritional outcomes. Model 2 includes potential covariates identified as being associated with both the exposure variables (cash and parenting support) and the outcome variables (nutritional status). Model 3 uses interaction terms to assess the potential multiplicative effects of cash and care on nutritional outcomes. Marginal effects models were also used to explore the additive effects of combined cash and care receipt on child nutrition outcomes. Probability predictions with 95% confidence intervals (CIs), adjusted for covariates, are presented. Covariates identified with strong associations (*p* < 0.2) with both predictor and outcome variables were included within the models. Covariates included were child gender, child age, child HIV status, number of household assets, and for the model exploring the association between cash and parenting support and child size, type of child home was also included in the model. Unadjusted and adjusted odds ratios (ORs and aORs, respectively) with 95% CIs are reported and *p*-values with thresholds of <0.05, <0.01, <0.001 were used.

## Results

Six hundred and twenty-four children and young adolescents lived in a household in receipt of a cash grant, while 230 received no cash grants. Table 1 below sets out comparison of socio-demographic and nutrition outcomes for those receiving cash grants compared to those not receiving cash grants. There were no gender differences according to grant receipt. Younger children and children from South Africa were significantly more likely to be in receipt of a grant (*t* = 3.74, *p* = 0.0002 and *χ*^2^ = 477.8, *p* < 0.001, respectively). One hundred and fifteen children in the sample (13.5%) were recorded as living with HIV. These children were significantly less likely to receive a grant than HIV-negative children (*χ*^2^ = 17.3, *p* < 0.001). On the nutritional variables, there were significant differences according to cash grant receipt on child-reported hunger, stunting, wasting, weight, food status, child size and child appearance (see Table 1).

**Table 1.** Descriptive characteristics of the sample and nutritional outcomes stratified by cash grant receipt.

	Total (n = 854) n(%)	Cash grant received (n = 624) n(%)	No cash grant received (n = 230) n(%)	χ2 or t, p-value
Gender				
Female	439 (52.3)	322 (73.4)	117 (26.7)	0.13, 0.72
Male	400 (47.7)	289 (72.3)	111 (27.8)	
Age (years)	*M* = 10.21 (SD 2.80)	*M* = 9.99 (SD 2.80)	*M* = 10.80 (SD 2.73)	3.74, 0.0002
Country				
South Africa	708 (82.90)	624 (88.14)	84 (11.86)	477.8, <0.0001
Malawi	146 (17.10)	0 (0.00)	146 (100.0)	
HIV status				
HIV positive	115 (13.47)	69 (60.00)	46 (40.00)	17.3, <0.0001
HIV negative/unknown	737 (86.30)	555 (75.31)	182 (24.69)	
Type of home				
Formal	689 (86.56)	481 (69.81)	208 (30.19)	13.47, <0.0001
Informal	107 (13.44)	93 (86.92)	14 (13.08)	
No. household assets	*M* = 3.90 (SD 1.93)	*M* = 4.38 (SD 1.58)	*M* = 2.60 (SD 2.16)	13.16, <0.0001
Went to bed hungry last night				
Yes	89 (11.18)	39 (43.82)	50 (56.18)	39.88, <0.0001
No	707 (88.82)	535 (75.67)	172 (24.33)	
Child stunted				
Yes	193 (23.34)	108 (55.96)	85 (44.04)	38.33, <0.0001
No	625 (76.41)	489 (78.24)	136 (21.76)	
Child wasting				
Yes	56 (6.82)	31 (55.36)	25 (44.64)	8.98, 0.003
No	765 (93.18)	565 (73.86)	200 (26.14)	
Child underweight				
Yes	33 (9.79)	20 (60.61)	13 (39.39)	7.71, 0.005
No	304 (90.21)	247 (81.25)	57 (18.75)	
Food status				
Child is well fed	515 (60.30)	438 (85.05)	77 (14.95)	135.8, <0.0001
Child eats regularly sometimes	210 (24.59)	90 (42.86)	120 (57.14)	
Child has less food to eat than needed	118 (13.82)	89 (75.42)	29 (24. 58)	
Child regularly has no food to eat	11 (1.29)	7 (63.64)	4 (36.36)	
Child small for size				
Yes	96 (11.24)	61 (63.54)	35 (36.46)	4.99, 0.03
No	758 (88.76)	563 (74.27)	195 (25.73)	
Child looks thin				
Yes	81 (9.48)	40 (49.38)	41 (50.62)	25.51, <0.0001
No	773 (90.52)	584 (75.55)	189 (24.45)	

From the data, 160 children and younger adolescents (20.3%) received neither cash nor parenting above the cut-off for good parenting. A further 501 children (63.5%) received either cash or parenting above the cut-off score. Finally, a group of 128 children (16.2%) received both cash transfers plus good parenting above the cut-off score.

Multivariable regression analyses showing associations of cash, good parenting and combined cash and good parenting with child nutrition outcomes were carried out, controlling for other predictors including child gender, age, HIV status, number of household assets and type of home. These data are set out in Tables 2 and 3.

**Table 2.** Logistic regression models exploring predictors of child nutrition outcomes.

	Went to bed not hungry last night (n = 720)	Child not stunted (n = 635)	Child not wasting (n = 777)	Child not underweight (n = 309)
	OR (95% CI)	OR (95% CI)	OR (95% CI)	OR (95% CI)
Model 1				
No cash, no care (*n* = 160)	1 (Ref)	1 (Ref)	1 (Ref)	1 (Ref)
Either cash or care (*n* = 501)	2.80 (1.74–4.52)***	2.59 (1.76–3.82)***	2.40 (1.31–4.39)**	2.61 (1.08–6.32)*
Cash plus care (*n* = 128)	7.14 (2.71–18.80)***	4.44 (2.43–8.12)***	2.94 (1.14–7.57)*	6.00 (1.52–23.70)**
Model 2				
No cash, no care	1 (Ref)	1 (Ref)	1 (Ref)	1 (Ref)
Either cash or care	1.59 (0.92–2.76)	2.09 (1.36–3.22)***	1.36 (0.68–2.71)	1.81 (0.68–4.84)
Cash plus care	3.75 (1.33–10.53)**	3.15 (1.64–6.04)***	1.29 (0.45–3.70)	4.10 (0.96–17.48)
Model 3				
No cash, no care	1 (Ref)	1 (Ref)	1 (Ref)	1 (Ref)
Cash	2.12 (1.17–3.84)*	2.38 (1.52–3.74)***	1.38 (0.66–2.90)	1.87 (0.68–5.17)
Care	0.77 (0.37–1.62)	1.23 (0.64–2.34)	1.29 (0.45–3.72)	1.52 (0.34–6.88)
Interaction – cash × care	2.53 (0.75–8.53)	1.12 (0.48–2.65)	0.73 (0.18–2.98)	1.44 (0.20–10.40)

OR: odds ratio; CI: confidence interval.

* *p* < 0.05, **p < 0.01, ***p < 0.001.

**Model 1:** Univariate regression analyses showing associations of cash grant receipt, good parenting and combined cash and good parenting with child nutrition outcomes.

**Model 2:** Multivariable regression analyses showing associations of cash grant receipt, good parenting and combined cash and good parenting with child nutrition outcomes controlling for other predictors: child gender (female), age (years), HIV status (HIV+), and number of household assets.

**Model 3:** Multivariable regression analysis showing the interaction between cash grant receipt and good parenting controlling for other predictors: child gender (female), age (years), HIV status (HIV+), and number of household assets.

**Table 3.** Logistic regression models exploring predictors of child nutrition outcomes using Child Status Index food security domain.

	Child has sufficient food (n = 515)	Child size appropriate (n = 758)	Child does not look thin (n = 773)
	OR (95% CI)	OR (95% CI)	OR (95% CI)
Model 1			
No cash, no care (*n* = 160)	1 (Ref)	1 (Ref)	1 (Ref)
Either cash or care (*n* = 501)	5.79 (3.87–8.66)***	1.42 (0.84–2.40)	3.54 (2.10–5.99)***
Cash plus care (*n* = 128)	11.77 (6.72–20.60)***	1.62 (0.77–3.40)	4.32 (1.84–10.16)***
Model 2			
No cash, no care	1 (Ref)	1 (Ref)	1 (Ref)
Either cash or care	3.38 (2.16–5.28)***	1.40 (0.78–2.53)	2.00 (1.09–3.67)*
Cash plus care	5.78 (3.16–10.60)***	1.60 (0.71–3.60)	2.15 (0.84–5.56)
Model 3			
No cash, no care	1 (Ref)	1 (Ref)	1 (Ref)
Cash	3.64 (2.30–5.76)***	1.54 (0.83–2.83)	2.07 (1.08–3.99)*
Care	2.34 (1.19–4.57)*	0.96 (0.41–2.26)	1.78 (0.68–4.64)
Interaction – cash × care	0.70 (0.30–1.60)	1.12 (0.38–3.32)	0.59 (0.16–2.16)

OR: odds ratio; CI: confidence interval.

*p < 0.05, **p < 0.01, ***p < 0.001.

**Model 1:** Univariate regression analyses showing associations of cash, good parenting and combined cash and good parenting with child nutrition outcomes.

**Model 2:** Multivariable regression analyses showing associations of cash, good parenting and combined cash and good parenting with child nutrition outcomes controlling for other predictors: child gender (female), age (years), HIV status (HIV+), and number of household assets. The analyses showing the association between cash receipt, good parenting and combined cash and good parenting with child size also controls for child’s type of home (living in informal housing).

**Model 3:** Multivariable regression analysis showing the interaction between cash grant receipt and good parenting controlling for other predictors: child gender (female), age (years), HIV status (HIV+), and number of household assets. The analyses showing the association between cash receipt, good parenting and child size also controls for child’s type of home (living in informal housing).

These tables set out both univariate and the multivariable regression analyses examining predictors for the seven nutrition measurements. With no cash and no care set as the reference category, receipt of either cash or parenting care was significantly associated with the child not being stunted, the child having sufficient food, and the child not looking thin. Three of the nutritional outcomes showed increased improvement amongst children receiving both cash transfers plus good parenting care above the cut-off. Rates of child-reported non-hunger increased from aOR: 1.59 (95% CI: 0.92–2.76) when receiving either cash or care, to aOR: 3.75 (95% CI: 1.33–10.53) when receiving both. Child non-stunting increased from aOR: 2.09 (95% CI: 1.36–3.22) to aOR: 3.15 (95% CI: 1.64–6.04), and parent-reported sufficient food from aOR: 3.38 (95% CI: 2.16–5.28) to aOR: 5.78 (95% CI: 3.16–10.60).

**Figure 1.**
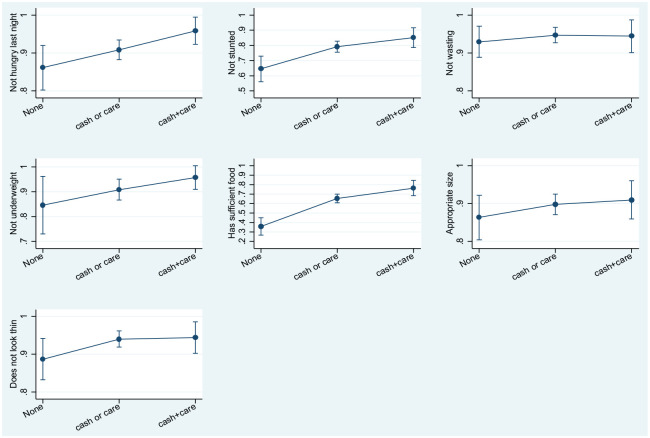
Marginal effects model testing for additive effects of cash and good parenting receipt on child nutrition outcomes. Adjusted for child gender, child age, child HIV status, number of household assets and, for the model exploring the association between cash and parenting support and child size, type of child home. None: no cash or care.

Controlling for all covariates, potential multiplicative effects of cash and care were explored using interaction terms in logistic regression models. The exponential coefficients (OR) of such interactions are presented. No statistically significant interactions were apparent, indicative of no multiplicative effects (see Tables 2 and 3). To explore potential additive effects of cash and care, estimates of the predicted probability of each nutritional outcome were calculated, controlling for all predictor variables (see Figure 1). Predicted probability of child-reported non-hunger was 86% when neither cash grant receipt nor sufficiently good parenting were received, 91% with either form of intervention, and upon receipt of both a cash grant and good parenting, 96% of children reported non-hunger. Similar patterns are shown for measures of non-stunting (65%, 79% and 85%, respectively), the child being of an appropriate weight (85%, 91% and 96%, respectively), caregiver reports of sufficient food access (36%, 65% and 76%, respectively), and the child being of an appropriate size (86%, 90% and 91% respectively). Predicted probability of child non-wasting was found to be 93% when neither cash grant nor sufficiently good parenting were received, 95% upon receipt of either a cash grant or good parenting and 94% upon receipt of a cash grant and good parenting. Predicted probability of having a child who looked to be of an appropriate size was 89% when neither a cash grant nor sufficiently good parenting were received, 94% upon receipt of either intervention, and remained at 94% when both interventions were received (see Figure 1).

## Discussion

This study uses a large sample of community-identified children and young adolescents in two countries in sub-Saharan Africa (South Africa and Malawi) to explore the impact of cash grant receipt and care provision (operationalised as good parenting) on child nutritional outcomes. Within the analyses, different levels of social protection (i.e. cash grant receipt/care provision) were explored: neither cash or care, either cash or care, and cash and care. Provision of social cash transfers and parenting support were found to be strongly and independently associated with improved child nutritional outcomes. Either cash or good parenting was a significant predictor of improved child status in five of the seven nutrition outcomes. Over and above these effects, combining cash plus care was a significant predictor for three of the seven nutrition outcomes. For some outcomes, either cash grants or good parenting was sufficient to improve the situation – with no added combination effect. The combination of provision did not enhance the positive effect for all measures, but did for three, notably child-reported hunger, child stunting and child food sufficiency.

The SDGs have rightly identified the need for more robust lenses to examine child development, more robust interventions to avoid silos, and more comprehensive planning to ensure maximising human potential. Our data show a clear possible pathway for utilising well-established interventions in concert with each other to extend the impact, enhance the reach and to allow for synergies in programming. When single interventions are not effective, there is a constant search for new novel interventions. However, our data suggest that well-tried interventions may well be effective and the novelty is providing them in combination.

The study is not without limitations. This study utilises cross-sectional data and future research may be necessary to explore these findings within longitudinal, more controlled and randomised designs. Our data are limited to two settings, and generalisation to other settings may need to be explored. Our parenting measures were a composite measure, and although these were solid within the evaluation, more robust and additional validated measures could be used in the future. Our data also focuses on children and younger adolescents, and it should be noted that the age range of the sample (5–15 years) focuses on different developmental periods (childhood and early adolescence). As such, the impacts of nutrition and good parenting may have differing effects across developmental periods. It is therefore important for future studies to explore effects across different developmental periods inclusive of infancy and childhood, younger and older adolescence.

However, these data suggest that the overall wellbeing of children can be greatly enhanced by combining two established social protection measures. When cash grant programmes are considered, a complex model should be envisaged where parenting interventions may help to supplement the efficacy of cash grants and impact on nutrition-related child outcomes. Some earlier studies have examined the relative benefits of child stimulation interventions on child outcomes and found these to be effective with long-term follow-up. These data would suggest that to optimise the impact, cash grants should be given in combination. Cash plus care seems to be a viable future pathway, specifically in areas of high deprivation, high poverty, and high HIV burden.
